# Urinary incontinence and related quality of life among elderly women in Tabas, South Khorasan, Iran

**DOI:** 10.1186/s12894-022-01171-9

**Published:** 2022-12-31

**Authors:** Zahra Najafi, Mohammad Ali Morowatisharifabad, Sara Jambarsang, Hassan Rezaeipandari, Roya Hemayati

**Affiliations:** 1 International Campus, Shahid Sadoughi University of Medical Sciences, Yazd, Iran; 2 Elderly Health Research Center, School of Public Health, Shahid Sadoughi University of Medical Sciences, Yazd, Iran; 3 Department of Aging Health, School of Public Health, Shahid Sadoughi University of Medical Sciences, Yazd, Iran; 4 Center for Healthcare Data Modeling, Departments of Biostatistics and Epidemiology, School of Public Health, Shahid Sadoughi University of Medical Sciences, Yazd, Iran; 5 Department of Internal Medicine, Shahid Sadoughi University of Medical Sciences, Yazd, Iran

**Keywords:** Prevalence, Urinary incontinence, Elderly women, Quality of life

## Abstract

**Background:**

Urinary incontinence (UI) is one of the most common problems in old age that is often seen in women, which causes not only physical problems but also psychological, social, economic problems and poor quality of life. The aim of the present study was to evaluate the UI and related quality of life (QoL) in elderly women.

**Methods:**

This cross-sectional study enrolled 369 women over 60 years old and living in Tabas city, Iran who were selected by cluster random sampling method. The instruments included the International Consultation on Incontinence Questionnaire-Short Form, the International Consultation on Incontinence Questionnaire Urinary Incontinence Quality of Life Module, and a demographic questionnaire. Data analysis was carried out using independent t-test, chi-square, and logistic regression in SPSS software.

**Results:**

The UI prevalence among participants was 24.9% and stress urinary incontinence was the most common type (40.2% of all elderly patients). The mean UI-related QoL score was 38.04 ± 11.67 from the score range of 22–76. There was a significant positive correlation between UI-related QoL score and UI score (r = 0.585, *p* < 0.001). Age, body mass index (BMI), constipation, history of cesarean section, hypertension, and the use of angiotensin receptor blockers are factors increasing the odds of having UI in this study population.

**Conclusion:**

Aging, some chronic diseases, high BMI, and the use of some drugs are related to UI prevalence. Also, it is associated with lower QOL among elderly women. Designing appropriate intervention programs, controlling chronic diseases, training in the proper use of drugs, and also some physical exercises can be effective in controlling and improving this common syndrome of old age and promoting their QoL.

## Introduction

Urinary incontinence (UI)—the complaint of any involuntary leakage of urine-is common in women that increases with age and may decrease quality of life [1]. Therefore, UI is one of the most common syndromes in old age, and depending on different populations, its prevalence has been reported between 2.8% in Nigeria and 57.7% in Iran [2]. The World Health Organization (WHO) has identified the UI as one of the priorities in the health field as impacts the quality of life of older people because it affects different aspects of life and leads to psychological, physical, and social consequences [3]. Negative psychological effects of UI include anxiety, worry, frustration, tension and stress, bad temper, low self-esteem, and self-confidence. The prevalence of depression and anxiety among people with UI is reported to be about 20–40% and 56%, respectively [4]. According to previous studies, the UI prevalence among older adults has been estimated between 30 and 70% [5–7]. The UI prevalence is different in different regions of Iran so the prevalence of this syndrome in the Iranian female community is reported between 20 and 60%, this wide range can be attributed to the methodology or the age range of the participants in different studies [8–11].

It is difficult to assess the true prevalence of UI for cultural and social reasons. This syndrome is commonly underreported because most elderly people regard it as a consequence of aging and may even feel embarrassed to express it [12]. The three most common types of UI in women are stress urinary incontinence (SUI), urge urinary incontinence (UUI), or a combination of both, mixed urinary incontinence (MUI) [13]. In people with SUI, complaints of involuntary loss of urine during coughing, sneezing, and other physical exertions (e.g., sporting activities) increase intra-abdominal pressure, whereas the person with UUI suddenly feels the need to urinate (loss of urine associated with urgency). MUI is a combination of the symptoms of SUI and UUI. Other types of UIs that require more specialized detection include overflow and functional UIs [14].

Previous studies have reported numerous factors for the incidence or exacerbation of UI, including aging [11, 15–17], female gender [16–18], childbirth [11, 19, 20], obesity [18, 19, 21, 22], chronic diseases [17, 18, 23, 24], menopause [25, 26], polypharmacy [17, 27, 28] and movement limitations [17, 18, 25].

One of the main UI consequences is a decrease in the quality of life (QoL) of older adults [27, 29]. Evidence shows that UI affects QOL in a variety of ways, as there are many differences in people's perceptions and responses to UI symptoms. First, people with UI usually have more chronic illnesses than non-affected ones. In addition, fluid intake, mobility, and diuretic therapy may affect the UI [29]. Therefore, UI has negative effects on various aspects of life, especially QOL and the physical, social and psychological dimensions of health [29, 30].

It is difficult to assess the true prevalence of UI for cultural and social reasons. This syndrome is commonly underreported because most elderly people regard it as a consequence of aging and may even feel embarrassed to express it [12]. The three most common types of UI in women are stress urinary incontinence (SUI), urge urinary incontinence (UUI), or a combination of both, mixed urinary incontinence (MUI) [13]. In people with SUI, complaint of involuntary loss of urine during coughing, sneezing, and other physical exertion (e.g., sporting activities) increase intra-abdominal pressure, whereas the person with UUI suddenly feels the need to urinate (loss of urine associated with urgency). MUI is a combination of the symptoms of SUI and UUI. Other types of UIs that require more specialized detection include overflow and functional UIs [14].

Previous studies have reported numerous factors for the incidence or exacerbation of UI, including aging [11, 15–17], female gender [16–18], childbirth [11, 19, 20], obesity [18, 19, 21, 22], chronic diseases [17, 18, 23, 24], menopause [25, 26], polypharmacy [17, 27, 28] and movement limitations [17, 18, 25].

One of the main UI consequences is a decrease in the quality of life (QoL) of older adults [27, 29]. Evidence shows that UI affects QOL in a variety of ways, as there are many differences in people's perceptions and responses to UI symptoms. First, people with UI usually have more chronic illnesses than non-affected ones. In addition, fluid intake, mobility, and diuretic therapy may affect the UI [29]. Therefore, UI has negative effects on various aspects of life, especially QOL and the physical, social and psychological dimensions of health [29, 30].

On the other hand, women are more susceptible to this syndrome due to their anatomical, social, and cultural conditions, as well as due to pregnancy and childbirth, and menopause. UI causes many serious problems for the patient physically, mentally, and socially. It may affect relatives even more severely, and at the same time, this syndrome imposes huge economic costs. Due to the importance of this common syndrome in older adults and the fact that there has been no study on the relationship between the prevalence of UI and QOL among older adults, the present study aimed to determine the status of UI and related QOL in elderly women of Tabas, South Khorasan Province, Iran.

## Methods

### Study design and participants

This cross-sectional study was conducted in Tabas city of South Khorasan province, Iran in 2020. The study population included 369 elderly women who were randomly selected. Inclusion criteria included age of 60 years and older living in urban areas of Tabas, and absence of cognitive problems based on the Persian version of the Mini-Mental State Examination (MMSE) test [31]. Exclusion criteria also included elderly women with deafness, severe disability, and lack of consent to participate in the study.

The required sample size was estimated at 369 people using 5% accuracy and the type I error = 0.05 using Cochran's formula. To select the participants, individuals were selected as a category in proportion to the number of people registered in the Iranian National Health System (INHS), in three urban of Tabas city (Kowsar, Golshan, and Golestan) after coordination with Tabas city health center. Tabas city has three comprehensive health centers. The total population of elderly women in Kosar, Golshan, and Golestan health centers is 879, 392, and 448 people, respectively. A total of 188, 85, and 96 people were randomly selected from Kowsar, Golshan, and Golestan health centers, respectively. They were invited to participate in the study and in person at healthcare centers. At first, the study purpose and method were explained to the participants, and after obtaining their consent, the questionnaires were completed in the form of face-to-face interviews in a private room where the elderly felt comfortable. The elderly women who were not able to come to the health center, the first author, referred to their homes and did the interviews for completing the questionnaires. All methods were carried out in accordance with relevant guidelines and regulations.

### Instruments

The first section of the questionnaire includes demographic and background information including age, level of education, place of residence, weight, height, smoking, employment status, history of abortion, marital status, living situation, disability status, and having chronic diseases or common problems. Weight and height were measured by standard protocol. Body Mass Index (BMI) is a person’s weight in kilograms (or pounds) divided by the square of height in meters (or feet). The next sections of the questionnaire included the following instruments.

#### International consultation on incontinence questionnaire-short form (ICIQ-UI SF)

The ICIQ-UI-short form is a brief and psychometrically robust patient-completed questionnaire that evaluates the frequency, severity, and QOL-related impacts of UI in men and women in research and clinical practice across the world. It contains six questions that investigate a person's condition over the past four weeks. Questions 1 and 2 are demographic questions and question 3 includes the frequency of UI, question 4 measures the amount of leakage, and question 5 measure UI-related QOL impact. The scores obtained from questions 3, 4, and 5 represent the actual score. Question 6 focuses on the time and type of leakage. The possible score range is 0–21 with higher scores indicating increased severity [32]. The reliability and validity of the Persian version of ICIQ-SF have been approved in a study by Hajebrahimi et al. [33].

#### International consultation on incontinence questionnaire urinary incontinence quality of life module (ICIQ-LUTSqol)

The ICIQ-LUTSqol [34] is a psychometrically robust patient-completed questionnaire evaluating the quality of life (QoL) in UI patients in research and clinical practice. The ICIQ-LUTSqol consists of 20 items and each item has two sections. Section "a" of each item is entered into the outcomes calculator and contributes to the overall score. Section "b" of each item is a visual analog scale to assess the bother score (score range:0–10), which does not contribute to the overall score but is intended as a guide to clinicians to indicate the overall bother experienced by the patient. Bother scores, including question 22, have not been entered into the outcome calculator. The possible score range is 19 and 76, with lower scores indicating a better QoL status. However, sections of b are assessed on a 0–10 scale, where 0 means no bother and 10 means maximum bother/maximum impact on section a, i.e. QoL [35]. The reliability and validity of the Persian version of ICIQ-LUTSqol have been evaluated and approved by Pourmomeny et al. [35].

### Statistical analysis

Data were collected and coded and then entered into SPSS and analyzed using descriptive tests. In descriptive statistics, mean, standard deviation, frequency and percentage, and minimum and maximum were used. Independent t-test and chi-square test were used to investigate the relationship between the studied variables and the UI, and the odds ratio of UI in terms of each variable when controlling other variables was determined using a logistic regression model.

## Results

The mean ± SD of participants' age was 70.86 ± 8.27 years (range of 60–95 years). The majority of the participants had elementary education (57.5%) and were married. Of them, 36.3% had joint pain. Approximately, 66% of participants suffered from hypertension. Also, 35.8% of them had hyperlipidemia and 30% had diabetes (Tables 2 and 3, total column).

The prevalence of UI among the participants was 24.9%. The mean UI score was 6.29 ± 2.75 from the score range of 3 to 15. The most common UI was SUI among participants (40.2%). The least common UI in both groups was nighttime incontinence or nocturnal enuresis (Table 1).

**Table 1:** Prevalence of UI types in the studied population and among the elderly with UI

Types of UI	Whole studied population (n = 396)		Elderly with urinary incontinence (n = 92)
	N	%	%
Urge	30	8.1	32.6
Nighttime	2	0.5	2.2
Stress	37	10	40.2
Mixed	11	3	12
Post-micturition Symptoms	5	1.4	5.4
With no reason	7	1.9	7.6
Total prevalence	92	24.9	

The mean ± SD of the UI-QoL score was 38.04 ± 11.67 from the score range of 22–76. There was a positive and significant correlation between UI-related QoL score and UI score (r = 0.585, *p* < 0.001). There was also no significant difference between different UI types in terms of UI-related QoL (Table 2).

**Table 2:** Mean and standard deviation of urinary incontinence-related quality of life score by types of urinary incontinence in the participants

	SUI	UUI	Nocturia	PMSUI	MUI	No obvious reason
ICIQ-LUTSqol Mean (SD)	38.13 (11.48)	35.20 (9.81)	38.50 (7.77)	33.20 (6.87)	44.36 (13.64)	47.85 (14.62)
*P*-value	0.052					

SUI = Stress Urinary Incontinence, UUI = Urge Urinary Incontinence, PMSUI = Post Micturition Symptoms Urinary Incontinence, MUI = Mixed Urinary Incontinence

There was a statistically significant relationship between the prevalence of UI types with age, history of cesarean section, lifestyle, and regular drug use (*p* < 0.05). The prevalence of UI was 30% in people aged 80 years and older and 26.2% in the participants without a history of cesarean section. Also, the prevalence of this syndrome was 35% among elderly women who lived with their single children and 28.2% among those who were taking medication (Table 3).

**Table 3:** UI prevalence according to some demographic variables in the participants

UI Variable		Yes		No		Total		*P*-value
		N	%	N	%	N	%*	
Age	60–69	65	23.4	213	76.6	278	75.3	0.04
	70–79	18	29.5	43	70.5	61	16.5	
	80 and above	9	30	21	70	30	8.1	
History of abortion	Yes	36	26.7	99	73.3	135	36.6	0.08
	No	56	23.9	178	76.1	234	63.4	
Level of Education	Illiterate	29	25	87	75	116	31.4	0.95
	elementary	52	28.6	160	75.5	212	57.5	
	Middle school and above	11	26.8	30	73.2	41	11.2	
Marital status	Married	62	23.8	199	76.2	261	70.7	0.42
	Unmarried	30	27.8	78	72.2	108	29.3	
History of cesarean section	Yes	5	13.5	32	86.5	37	10	0.03
	No	87	26.2	245	73.8	332	90	
Employment status	Outdoor job	2	100	0	0	2	0.5	0.15
	Housewife	83	24.4	257	75.6	340	92.1	
	Retired	7	25.9	20	74.1	27	7.3	
Life style	With husband	62	23.6	201	76.4	263	55	0.03
	With single children	7	35	13	65	20	21.6	
	With married children	4	25	12	75	16	3	
	Alone	19	27.1	51	72.9	70	11.6	
Medication use	Yes	87	28.2	222	71.8	309	83.7	0.001
	No	5	8.3	55	91.7	60	16.3	
*Total percent								

There was also a statistically significant relationship between the prevalence of UI with cardiovascular diseases, respiratory diseases, arthritis and bone diseases, constipation, imbalance, joint pain, hypertension, digestive problems, sleep problems, and anorexia (*P* < 0.05) (Table 4).

**Table 4:** UI prevalence according to history of common diseases in the participants

Common diseases/problems		UI		*P*-value
		N	%	
Cardiovascular diseases	Yes	29	37.2	0.005
	No	63	21.2	
Diabetes	Yes	30	27	0.314
	No	62	24	
Depression	Yes	12	38.7	0.081
	No	80	23.7	
Respiratory diseases	Yes	11	55	0.003
	No	81	23.2	
History of stroke	Yes	0	0	0.331
	No	92	25.3	
Arthritis and bone diseases	Yes	18	47.4	0.001
	No	74	22.4	
Cancer	Yes	1	50	0.430
	No	90	24.6	
Constipation	Yes	14	45.2	0.009
	No	78	23.1	
Multiple sclerosis	Yes	1	16.7	0.53
	No	91	25.1	
Imbalance	Yes	18	50	0.001
	No	74	22.2	
Joint pain	Yes	49	36.6	0.001
	No	43	18.3	
Hypertension	Yes	70	29	0.016
	No	22	17.2	
Digestive problems	Yes	17	37.8	0.043
	No	55	23.1	
Sleep problems	Yes	30	39.5	0.002
	No	62	21.2	
Anorexia	Yes	10	47.6	0.019
	No	82	23.6	

According to the results of raw model regression analysis, the odds ratio of developing UI in elderly women with constipation was 2.7 times higher than those without constipation. Also, the odds ratio of developing UI increased by 6% with a one-unit increase in BMI. The odds ratio of developing UI in the elderly with hypertension was almost twice. According to the results of regression analysis in the adjusted model based on age, BMI, constipation, cesarean section, hypertension, and angiotensin receptor blockers, the odds ratio of developing UI in elderly women with constipation were 2.6 times higher than those without constipation. Also, the odds ratio of developing UI increased by 6% with a one-unit increase in BMI (Table 5).

**Table 5:** UI risk factors using multiple logistic regression model (model adjusted based on age, body mass index, constipation, cesarean section, hypertension and angiotensin receptor blocker use)

Variables	Estimation of coefficients	The standard error	*P*-value	Odds ratio (OR)	95% confidence interval	
					Lower limit	Upper limit
Constipation	0.973	0.42	0.021	2.64	1.15	6.05
History of cesarean section	− 0.944	0.52	0.06	0.38	0.14	1.07
Age	0.031	0.01	0.11	1.032	0.99	1.07
BMI	0.62	0.02	0.029	1.06	1.00	1.12
Number of deliveries	− 0.031	0.056	0.58	0.96	0.86	1.08
Hypertension	0.031	0.37	0.93	1.03	0.49	1.16
Taking angiotensin receptor blocker	− 0.074	0.31	0.81	0.92	0.49	1.72

## Discussion

UI is one of the most common problems in old age that not only causes physical problems but also causes psychological, social, and economic problems as well as poor quality of life. The aim of the present study was to determine the status of UI and UI-related QoL in elderly women living in Tabas, South Khorasan Province, Iran. The prevalence of UI in elderly women was 24.9%. The UI prevalence was 33% and 25% in Amirkola city, Mazandaran province [8], and Khorasan, respectively [9]. The overall UI prevalence in Iranian women is estimated at 46% [10]. The prevalence of UI varies by country and in elderly women in the world was 37.1% based on a meta-analysis [36]. It can be stated that wide differences in the UI prevalence can be due to differences in research methods used, different cultures or races of study populations or the existence of different predisposing factors in those areas. Also, after observing the prevalence in different regions, it can be stated that the UI prevalence is quite diverse in different populations, which can be due to cultural and social differences. Estimating the UI incidence rate requires proven definitions of UI to prevent misinterpretation. It is shown that the different UI incidence rate in women is largely due to different UI definitions in each study. It also explains our problem when comparing the present data with the existing literature [37]. Following the current ICS definition, we used a validated questionnaire to standardize the UI definition and its diagnosis.

The most common type of UI in the present study was SUI which is consistent with the results of many studies [10, 11, 19, 38]. SUI following an increase in abdominal pressure is to such an extent that it overcomes the bladder blockage pressure. Abdominal pressure increases when coughing, sneezing, laughing, climbing stairs, or lifting objects. SUI is more prevalent among women due to pregnancy and childbirth, and also the decrease in estrogen production during the postmenopausal period also leads to muscle atrophy and consequent UI [39]. Also, the higher SUI incidence can also be due to various factors [10]. However, there are studies that have reported that other types of UI are more prevalent. For example, Pathiraja et al. [40] reported that UUI was more prevalent and Agarwal et al. also showed that UUI and mixed UI were more common [41] were the most common.

The results showed a statistically significant relationship between the UI prevalence with age, history of cesarean section, lifestyle, and drug use. That is, the UI prevalence was 30% in people aged 80 years and older and 26.2% in elderly women with a history of cesarean section. Also, the prevalence of this disorder was 35% among the elderly who lived with their single children. The majority of previous relevant studies revealed that UI prevalence in women increased with age [11, 19, 37, 42]. With age, anatomical and physiological changes of the urogenital system such as muscle and axon degeneration, decrease in bladder capacity, increase in detrusor activity and consequent decrease in detrusor contractile strength occur and as a result, elderly women are prone to UI [43]. Overall, increased UI prevalence in elderly women may be due to the relaxation of the pelvic floor muscles and connective tissue supporting the urethra.

Only 26% of the participants who had a history of cesarean section reported that they are suffering from UI, and the prevalence of UI was higher among women who had no history of cesarean section. The results of previous studies also have shown that vaginal delivery may be related to UI and not cesarean Section [44, 45]. Some other studies have not reported a definitive relationship between the mode of delivery and UI [46].

There was a statistically significant relationship between the prevalence of UI and drug use. The results of other studies also indicate the relationship between drug use and UI prevalence [8, 17, 27, 28]. “In principle, drugs could cause incontinence by lowering bladder outlet resistance and/or by increasing intravesical pressure, which disrupts the normal pressure relationship between the bladder and urethra and leads to urinary leakage; other possibilities include disturbances of the central nervous control of voiding or an overproduction of urine [47].”

The highest prevalence of UI was reported in the elderly with respiratory diseases was imbalance, anorexia, arthritis and bone diseases, and constipation, which is consistent with the results of other studies. Studies on the relationship between various diseases and UI have identified chronic respiratory patients as a risk factor for UI [17, 23, 48]. Increased abdominal pressure in chronic respiratory diseases associated with chronic cough, increases the pressure on the urethra, which in turn affects the supporting tissues of the pelvis, which ultimately can cause a UI in the individual. Constipation, arthritis, rheumatism, imbalance, and heart disease in various studies have been considered as risk factors for UI [8, 24, 49–51]. Constipation is strongly associated with UI due to high abdominal pressure, which increases urethra pressure and affects the pelvic supporting tissue. A fecal impaction can change the position of the pelvic organs and put pressure on the bladder, thereby reducing its ability to hold urine. UI also occurs due to an overactive bladder, urinary retention, and loss of sphincter control due to constipation [52, 53].

According to the results of regression analysis in the adjusted model based on age, BMI, constipation, cesarean section, hypertension and use of angiotensin receptor blockers, the odds ratio of developing UI in the women with constipation was 2.6 times higher than those without constipation. Also, the odds ratio of developing UI increases by 6% with a one-unit increase in BMI. The results of the present study are consistent with the results of other research. For example, in a meta-analysis of the relationship between constipation and UI risk [53], results showed that constipation was a major risk factor for UI. Obesity and high BMI [18, 19, 21] and chronic diseases [17, 18, 23, 24] are other predictors and risk factors for UI in elderly women.

UI-related QoL was low and had a significant positive correlation with the UI score. There was also no significant difference in the UI- related QoL in the affected elderly based on the types of UI. Coyne et al. [30] also reported poorer QoL in elderly people with SUI and mixed UI. The results of most studies indicate a relationship between UI and QoL and the elderly people had poorer QoL [19, 37, 41, 54]. People with UI usually have more chronic illnesses than non-affected ones. Many patients are reluctant to seek medical help or treatment because they think the disease is incurable, and feeling ashamed or scared. These patients usually avoid meeting friends, traveling, and participating in other social activities. As a result, they experience social isolation, psychological and physical consequences, and subsequent poor QoL [27, 55].

## Conclusion

The prevalence of UI indicates this is a common condition and SUI is the most common type of IU among older women in the Iranian community that should be addressed by health care centers. Interventional programs for elderly people and their families about UI, risk factors such as aging, some chronic diseases, high BMI, and the use of some drugs and their management are essential for promoting their QoL.

## Study limitations

The present study has been performed on a number of elderly women in Tabas, therefore, the present study imposes limitations in the field of generalizations, interpretations, and etiological citations of the studied variables due to its study population and the methodology that should be considered. The impossibility of matching the members of the research group in terms of variables, which affected the structures studied in the present study, was another limitation of the present study. For example, the participants were not selected from families with the same socioeconomic status. Elderly women may not reflect their true feelings when answering questions for reasons and attempts were made to minimize this limitation by emphasizing the confidentiality of the information sought. It is likely that the elderly’s situation affected their responses when filling out the questionnaires, which was beyond the researcher's control. All data were collected via patient interview (not patient recall by independently completing the surveys or by consulting with the medical record). Additionally, the educational level attained by the population is low. For these reasons, should be cautious about interpreting relationships between the findings.

## Data Availability

The datasets used and/or analysed during the current study are available from the corresponding author on reasonable request.
